# Pulsed Magnetic Stimulation for Stress Urinary Incontinence and Its Impact on Sexuality and Health

**DOI:** 10.3390/medicina58121721

**Published:** 2022-11-24

**Authors:** Pablo González-Isaza, Rafael Sánchez-Borrego, Félix Lugo Salcedo, Nuria Rodríguez, Diana Vélez Rizo, Irene Fusco, Silvia Callarelli

**Affiliations:** 1 Ginecología y Obstetricia, Centro Médico Teknon, 08017 Barcelona, Spain; 2 Ginecología y Obstetricia, Ginecología Avanzada, Bogotá 110311, Colombia; 3 El.En. Group, 50041 Calenzano, Italy; 4 Studio Medicò Surgical Laser, 51017 Pescia, Italy

**Keywords:** magnetic field therapy, pelvic floor dysfunction, stress, urinary incontinence quality of life, sexuality

## Abstract

It is becoming increasingly common that patients’ preferences move towards non-surgical approaches, such as pulsed magnetic stimulation, for female stress urinary incontinence. Objective: We evaluated the efficacy and safety of a device that uses electromagnetic technology to treat urinary incontinence, with an emphasis on health-related quality of life. Methods: A total of 47 female subjects from 18 to 80 years old were enrolled. After block randomization, treatment consisted of 2 pulsed planar magnetic stimulation sessions per week for 4 weeks (8 sessions). Validated questionnaires: Female Sexual Function Index, International Consultation on Incontinence Questionnaire for Urinary Incontinence: Short Form, and Pelvic Floor Bothersome. Follow-ups were performed at weeks 1, 9, and 14. Results: The present study is one of the first clinical trials published evaluating the efficacy and safety of the electromagnetism-based device with flat configuration in patients with stress urinary incontinence, showing a reduction in PFBQ, ICQSF, and Oxford test scores during follow-up, and significantly at week 14 of follow-up, which implied a favorable impact on clinical outcomes, quality of life, and sexuality. Conclusions: The improved results in the treatment group compared with the simulated group show that pulsed magnetic stimulation is a safe and attractive non-invasive alternative for patients who prefer non-surgical treatments.

## 1. Introduction

Weakness of the pelvic floor muscles (PFM) causes lower urinary tract symptoms (LUTS), such as loss of bladder control, genital prolapse, and decreased sexual function, which have a substantial impact on women’s physical and mental health, as well as their quality of life (QOL) [1]. Pelvic organ prolapses, fecal incontinence, and urine incontinence symptoms are all frequent signs of pelvic floor dysfunction. Although the actual prevalence is unclear, urine incontinence (UI) might affect at least one in four women throughout the course of their lives [2,3].

Most pelvic floor disorders are treated first with behavior modification, weight loss, and pelvic floor physical therapy [4]. Physiotherapy has been shown to be effective in treating pelvic floor disorders, with symptoms improving in up to 70% of UI patients [5]. Unfortunately, not all patients can or want to receive pelvic floor physical therapy. In order to assist rehabilitation and perineal muscular self-awareness, patients undress during treatment, and the therapist works by internally palpating the vaginal and rectum muscles. These traditional approaches may be troublesome with regards to patient adherence due to the invasiveness and discomfort generated by using electrodes inside the vagina.

Magnetic stimulation (MS) is a novel approach that has been approved by the Food and Drug Administration (FDA) as a conservative treatment for UI since 1998, and it has already shown effective results in previous studies with encouraging long-term response rates [6]. It is becoming a popular non-invasive alternative and attractive surgical procedure for patients who do not wish to undergo invasive procedures [7]. High-intensity focused electromagnetism is utilized in MS treatment to activate the PFM motor neuron, resulting in longer, supramaximal contractions and the activation of more muscle fibers, which are comparable to 12,000 regular contractions with Kegel pelvic floor exercises [8,9]. It has various benefits, such as little adverse effects, automated contractions (patients do not need to know which muscles to contract), improved comfort as patients can remain completely clothed, and simplicity of administration, which greatly alters patients’ perceptions of the treatment’s efficacy. Due to the non-thermal nature of MS technology, any risk of thermal tissue damage is eliminated.

Based on these scientific findings, the purpose of the current study was to assess the efficacy and safety of a device that uses an innovative electromagnetic technology to treat urinary incontinence with an emphasis on health-related quality of life.

DR ARNOLD (DEKA M.E.L.A, Florence, Italy) is a device approved by the European Commission (EC) for non-invasive flat electromagnetic stimulation of the PFM to rehabilitate weak pelvic muscles and restore neuromuscular control for women with UI. At the same time, patients remain comfortably dressed and seated under ergonomic conditions.

## 2. Materials and Methods

The clinical setting was located inside the Aesthetic Functional and Regenerative Gynecology Unit of Teknon Medical Center, Barcelona, Spain, between September 2021 and April 2022. Ethics approval was obtained through the Comité Ético de Investigación con Medicamentos (CEIM) at the Hospital Universitari General de Catalunya, Barcelona.

Patient’s inclusion criteria were the following: age range (years) 18–80; informed consent; BMI (Kg/m^2^) < 30.0; SUI documented by stress test; willing to participate; adherence to the protocol; QOL impact related to SUI.

Patient’s exclusion criteria were the following: previous treatment with energy-based devices (EBD); pelvic organ prolapse quantification (POPQ) > Stage II; previous pelvic floor repair; pregnancy; metallic implants or pacemakers; pessary use; post void residual volume (mL) > 100.

A complete medical history and urogynecological examination were performed, including the Oxford test. Recruited patients were asked to complete the following validated questionnaires: Female Sexual Function Index (FSFI) [10], International Consultation on Incontinence Questionnaire for Urinary Incontinence: Short Form (ICQSF) [11], Pelvic Floor Bothersome Questionnaire (PFBQ) [12]. Patients were also asked to complete a 3-day bladder diary before the first session and at the 14-week follow-up. With the use of the 24 h voiding diary, one can check specifically for leak episodes, urgency episodes, and the use of pads.

After 4 sessions, 8 sessions, and up to 14 weeks of follow-up, the previously described tools were used to evaluate important changes from baseline to the end of the protocol. Secondary response included improvement in QOL measures.

For treatment, the patients were seated and fully clothed on the device (DR ARNOLDS, DEKA M.E.L.A, Florence, Italy). The protocol Hypotonus/Weakness 1 was selected for this study, and 8 sessions of 30 min were performed by patients twice a week. A registered nurse adjusted the method of treatment and the positioning of patients according to the clinical manual indications.

The same protocol used for the treatment group was also used for the control group, but with a maximum emission intensity of 5%. This intensity allows the patient to perceive a vibration that simulates the functionality of the system, but with values that are not adequate to achieve an effective physical exercise, while at the same time giving the patient the perception of the delivery of the magnetic stimulus. To induce muscle contraction, the system uses a sequence of emissions with an intensity that can be set between 0% and 100% of the maximum value allowed. The system also, during the emission, due to the particular generation of the magnetic field, emits unequivocal noises that determine its operating status. Finally, a standard protocol provides minimum intensity levels of at least 30% to induce a supra-maximal contraction.

Once the consent forms had been signed and the baseline visits had been completed, the participants were randomized in to one of the two comparison groups (treatment group and simulation group). Randomization was completed using computer generated random numbers (simple randomization). Participants were randomly assigned to groups in a 1:1 ratio. The procedure was performed by a qualified nurse who as not part of the study.

The investigator/sub-investigators were not told which group they had been assigned to. Likewise, patients did not know in which group they had been included (treatment or simulation).

For statistical analysis, the ANOVA test of repeated measures was performed to evaluate the change in the score of the applied questionnaires. Data were represented as means ± standard deviation (SD). Statistical significance was considered achieved when *p* < 0.05.

## 3. Results

**Figure 1:**
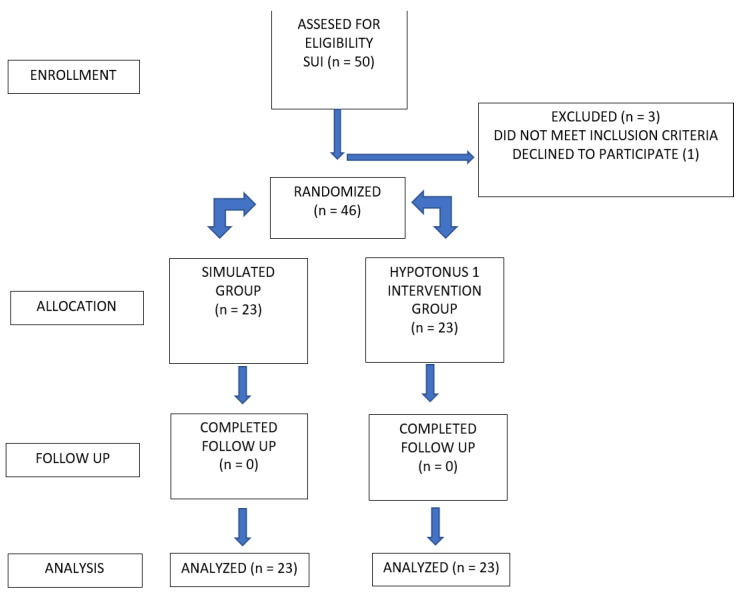
Flow chart of patient’s study enrollment, allocation and follow up.

**Figure 2:**
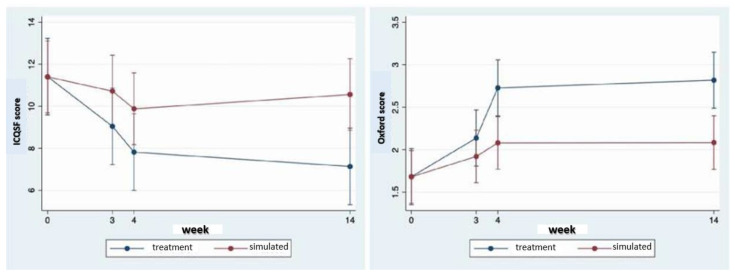
ICQSF and Oxford questionnaire scores at weeks 3, 4, and 14 of follow-up.

Forty-seven female patients were included (22 in the treatment group and 25 in the simulated group) with a mean age of 54.17 ± 11.42 years, mean reported parity of 1.74 ± 0.73 births, and mean body mass index (BMI) of 24.27 ± 3.24 Kg/m^2^. All patients satisfied the criteria for stress urine incontinence (SUI) (Figure 1) and 24 of these patients (50%) were in a state of menopause. The study groups were homogeneous in most of the variables analyzed (*p* > 0.05). In the treatment group, there was a higher proportion of patients diagnosed with mild incontinence. In contrast, in the simulated group, the majority of patients were diagnosed with moderate incontinence without statistically significant differences. Regarding the baseline measurements reported for the PFBQ, ICQSF, and Oxford questionnaires, there were no significant differences between the randomized groups. In the case of the FSFI questionnaire, significantly higher scores were reported in the treatment group patients than in the simulated group, all being below a score of 26, which is indicative of sexual dysfunction (Table 1). In the case of the PFBQ questionnaire, patients randomized to the treatment group reported lower scores at all cut-off points. At week 14 of follow-up, there was a significant decrease compared to the simulated group (*p* = 0.006). For the ICQSF questionnaire, for the treatment group, a reduction was observed throughout the follow-up time and a significant reduction compared to the simulated group at week 14 (*p* = 0.006) was observed. In the case of the FSFI questionnaire, patients in the treatment group reported significantly higher scores compared to the simulated group at week 14 of follow-up (*p* = 0.041). Finally, for the Oxford questionnaire, significantly lower scores were found for the patients in the treatment group versus the simulated group at week 4 (*p* = 0.007) and week 14 of follow-up (*p* = 0.010) (Table 2, Figure 2).

## 4. Discussion

The system generates a selective supra-maximal activation of the muscle unit. By targeting neuromuscular tissue and producing electric currents, this method induces strong PFM contractions. Neurons are depolarized by electric currents, which results in concentric contractions and a lifting of all PFM [13]. Electromagnetic radiation, deep penetration, and stimulation of the entire pelvic floor area are crucial to its effectiveness. By promoting more effective myofibril growth, which results in muscle fiber hypertrophy, the synthesis of additional protein strands, and muscle fiber hyperplasia, this procedure has a direct impact on muscle structure. An advantage of the device is its greater homogeneity of magnetic field distribution in a broader area, which allows greater recruitment of muscle fibers without creating areas of variable stimulation intensity.

Top flat magnetic technology stimulates deep PFM and restores neuromuscular control. Maximal voluntary contraction (MVC) is the most tension that a muscle can create and retain physiologically, however it is generally just for a fraction of a second. Supramaximal contractions have a tension greater than MVC. This technique is capable of producing supramaximal PFM contractions and holding them for a few seconds. These contractions are not controlled by the brain and directly affect the peripheral nerves in the pelvic floor region. This phenomenon of supramaximal contractions is normally impossible to induce by voluntary muscular movement (e.g., Kegel exercise). The secret to this technology’s efficacy lies in the electromagnetic fields’ steadily rising strength and pulse frequencies, which provide the unique vigorousness of the contractions [14].

Guidelines for the nonsurgical therapy of UI were published by the European Association of Urology in 2017. Treatment options include pelvic floor muscle training (PFMT), bladder training (BT), electrical stimulation, MS, and posterior tibial nerve stimulation [15]. Our results reproduce the previous findings of Lo et al. [16] regarding QOL improvement after a complete protocol of extracorporeal MS. However, in the case of the FSFI score, it was considered that from the beginning of the randomization, there were significant differences between the scores obtained between the groups analyzed, which may be related to an inadequate/unequal selection of the patients affecting the results obtained. Additionally, from the beginning of the experiment, all the patients reported scores below 26, indicating that the patients presented sexual dysfunction before being evaluated. Despite this, the treatment with the device showed less significant worsening compared to the simulated group, which may be related to a better QOL and sexuality of the patients included in this study. We consider that when carrying out the intervention and applying the questionnaires, it is likely that most of the patients would have tried to increase their sexual activity and that some decreases in the scores of the simulated group may be related to the fact that the act of sitting in a chair, confronting the operator, and talking about the pelvic floor, already offers by itself an effect on psychosocial rehabilitation and improvement of SUI. 

Regarding the treatment specifications, Lim et al. [8] performed a systematic review describing the basic principles of magnetic field stimulation and the potential therapeutic options for SUI. It was clearly described that the parameters should be adjusted for each type of incontinence. In their findings, it was suggested that approximately 50 Hz were required to achieve a good pelvic floor contraction for the treatment of SUI [8]. In contrast, our study used a protocol named “Hypotonus weakness 1”, composed of ascending frequencies up to 30 Hz. According to our results, this was enough to improve SUI without generating discomfort for our patients. 

The main strength of our study is the comparison with a simulated group that received a simulated treatment with around 5% of the total energy without generating any muscular stimulation or effects. It was demonstrated that the treatment group had pronounced statistically significant effects regarding improvement of SUI. Furthermore, a clinical evaluation was correctly performed in all patients as well as a subjective outcomes evaluation using standardized questionnaires. Furthermore, past evaluations had either short-term or no follow-up, since several researchers questioned the long-term effectiveness of MS on UI. Despite this, our study showed promising results at week 14 of follow-up. Accordant with our results, Ünsal et al. [17] reported an improvement of 79% of SUI urodynamic evaluation after the treatment protocol demonstrated an increase in mean Valsalva leak point pressure (VLPP) from 87.3 ± 15.9 to 118.0 ±11.0 cm, and Pad test weight was reduced from 15.4 ± 11.0 to 5.8 ± 7.3 g in the stress group (*p* = 0.000) at year 1 of follow-up. Also, the clinical prospective non-randomized study carried out by Lukanovic et al., 2021 [18] demonstrated the success rate of using MS in treating UI with ICIQ-UI SF questionnaires.

In addition, the recent published study of Braga et al. shows the efficacy of the 3 Tesla FMS technology, both in patients with pure stress urinary incontinence (SUI) and in women with pure overactive bladder (OAB) symptoms, with excellent outcomes [19].

Finally, the technology we used is also an educative device, which improves the awareness of muscle tone in the patients; this aspect is strictly related to the success of the treatment, which has also been reported by Gilling et al. [20] inside a double-blind, randomized controlled trial experiment comparing simulated therapy with pelvic floor electromagnetic stimulation for the treatment of women with SUI. At 8 weeks for the 20-min pad test and the number of pads used daily, the authors discovered a statistically significant improvement in the treatment group, notably in women with poor voluntary control of the pelvic floor muscle [21].

The noninvasiveness properties of this novel treatment allow the patients to adhere better to the protocol by undergoing touch-ups or repeated sessions with intervals defined by individual clinical conditions. Due to the great potential of this treatment, it has also been used to treat hypertonicity of PFM; lower frequencies around 10 Hz are used to relax the muscles and alleviate the pain associated; Biondo et al. [21] in recent research, found statistically significant changes in 34 subjects who met the criteria for pelvic floor pain due to hypertonicity.

In addition, recent published results [22] suggest that this technology could be used as an alternative and convenient male UI treatment tool.

### Study Limitations

The weaknesses of this study may be represented by the limited sample size and the lack of objective evaluation. Furthermore, we plan to extend the follow-up period in further future studies.

## 5. Conclusions

SUI is a highly prevalent condition. Unfortunately, effective therapies are limited. Data from short follow-up studies show improvement with PFM rehabilitation programs to date, considered to be the gold standard intervention for UI. Unfortunately, adherence to this type of intervention is very low due to its invasiveness. Top flat MS has shown to be safe and effective in previous studies; other advantages include no adverse effects. The encouraging improvement of SUI in the treatment group compared to the simulated group shows that pulsed MS is a safe and attractive non-invasive alternative for patients who prefer non-surgical treatments. Data was conclusive regarding the score improvement for all the questionnaires from the baseline up to 14 weeks of follow-up.

## Figures and Tables

**Table 1:** General characteristics of the patients according to the assigned treatment.

Variable	Total *n* = 47	Treatment Group *n* = 22	Simulated Group *n* = 25	*p* Value ^a^
	Mean (SD)	Mean (SD)	Mean (SD)	
Age (years)	54.17 (11.42)	53.63 (12.32)	54.64 (10.81)	0.767
Parity	1.74 (0.73)	1.86 (0.63)	1.64 (0.81)	0.303
BMI (Kg/m^2^)	24.27 (3.25)	23.97 (3.31)	24.53 (3.25)	0.562
Incontinence ^δ^				0.096
Mild	26 (55.32)	15 (68.18)	11 (42.31)	
Moderate	21 (44.68)	7 (31.82)	14 (66.67)	
PFBQ (initial)	12.78 (8.29)	10.54 (5.38)	14.76 (9.88)	0.082
ICQSF (initial)	11.40 (4.31)	11.41 (4.23)	11.40 (4.46)	0.994
FSFI	21.19 (8.64)	24.39 (8.02)	18.38 (8.31)	0.015 *
Oxford test	1.68 (0.69)	1.68 (0.56)	1.68 (0.80)	0.993

BMI = Body Mass Index; PFBQ = Pelvic Floor Bothersome Questionnaire; ICQFS = International Consultation on Incontinence Questionnaire for Urinary Incontinence-Short Form; FSFI = Female Sexual Function Index. ^a^ *p* value attained from Student’s *t*-test. ^δ^ Absolute frequency and percentage; Chi-square test. * Statistically significant (*p* < 0.05).

**Table 2:** Variation in the scores of the PFBQ, ICQSF, FSFI, and Oxford questionnaires at weeks 3, 4, and 14 of follow-up.

Variable	Total *n* = 47	Treatment Group *n* = 22	Simulated Group *n* = 25	*p* Value ^a^
	Mean (SD)	Mean (SD)	Mean (SD)	
PFBQ 3 weeks of follow-up	9.93 (4.34)	9.04 (3.87)	10.72 (4.65)	0.190
PFBQ 4 weeks of follow-up	8.91 (4.57)	7.81 (4.33)	9.88 (4.63)	0.124
PFBQ 14 weeks of follow-up	8.95 (4.43)	7.13 (3.65)	10.56 (4.51)	0.006 *
ICQSF weeks of follow-up	9.93 (4.34)	9.04 (3.87)	10.72 (4.65)	0.190
ICQSF 4 weeks of follow-up	8.91 (4.57)	7.81 (4.33)	9.88 (4.63)	0.124
ICQSF 14 weeks of follow-up	8.95 (4.43)	7.13 (3.65)	10.56 (4.50)	0.006 *
FSFI 3 weeks of follow-up	19.98 (10.01)	21.51 (9.70)	18.64 (10.28)	0.330
FSFI 4 weeks of follow-up	15.96 (9.35)	17.51 (10.02)	14.61 (8.71)	0.294
FSFI 14 weeks of follow-up	20.19 (9.53)	23.19 (9.95)	17.56 (8.48)	0.041 *
Oxford 3 weeks of follow-up	2.02 (0.73)	2.13 (0.77)	1.92 (0.70)	0.320
Oxford 4 weeks of follow-up	2.38 (0.84)	2.72 (0.76)	2.08 (1.74)	0.007 *
Oxford 14 weeks of follow-up	2.44 (0.95)	2.81 (0.85)	2.12 (0.92)	0.010 *

PFBQ = Pelvic Floor Bothersome Questionnaire; ICQFS = International Consultation on Incontinence Questionnaire for Urinary Incontinence-Short Form; FSFI = Female Sexual Function Index. ^a^ *p* value attained from Student’s *t*-test. * Statistically significant (*p* < 0.05).

## Data Availability

Data available on request due to privacy restrictions.
